# Laser-Induced Graphene on Additive Manufacturing Parts

**DOI:** 10.3390/nano9010090

**Published:** 2019-01-11

**Authors:** Lishi Jiao, Zhong Yang Chua, Seung Ki Moon, Jie Song, Guijun Bi, Hongyu Zheng, Byunghoon Lee, Jamyeong Koo

**Affiliations:** 1 Singapore Centre for 3D Printing, School of Mechanical and Aerospace, Nanyang Technological University, Singapore 639798, Singapore; jiao0011@e.ntu.edu.sg (L.J.); chua0735@e.ntu.edu.sg (Z.Y.C.); 2 Singapore Institute of Manufacturing Technology, 73 Nanyang Drive, Singapore 637662, Singapore; so0001ie@gmail.com (J.S.); gjbi@simtech.a-star.edu.sg (G.B.); 3 School of Mechanical Engineering, Shandong University of Technology, Zibo 255000, China; zhenghongyu@sdut.edu.cn; 4 Global Technology Center, Samsung Electronics Co., Ltd., Suwon 16677, Korea; bh13.lee@samsung.com (B.L.); jamyeong.koo@samsung.com (J.K.)

**Keywords:** 3D printing, additive manufacturing, laser direct writing electronics, laser-induced graphene

## Abstract

Additive manufacturing (AM) has become more prominent in leading industries. Recently, there have been intense efforts to achieve a fully functional 3D structural electronic device by integrating conductive structures into AM parts. Here, we introduce a simple approach to creating a conductive layer on a polymer AM part by CO_2_ laser processing. Scanning electron microscopy (SEM), transmission electron microscopy (TEM), and Raman spectroscopy were employed to analyze laser-induced modifications in surface morphology and surface chemistry. The results suggest that conductive porous graphene was obtained from the AM-produced carbon precursor after the CO_2_ laser scanning. At a laser power of 4.5 W, the lowest sheet resistance of 15.9 Ω/sq was obtained, indicating the excellent electrical conductivity of the laser-induced graphene (LIG). The conductive graphene on the AM parts could serve as an electrical interconnection and shows a potential for the manufacturing of electronics components. An interdigital electrode capacitor was written on the AM parts to demonstrate the capability of LIG. Cyclic voltammetry, galvanostatic charge-discharge, and cyclability testing demonstrated good electrochemical performance of the LIG capacitor. These findings may create opportunities for the integration of laser direct writing electronic and additive manufacturing.

## 1. Introduction

Additive manufacturing (AM), also known as 3D printing, is playing an increasingly significant role in various industries, including automotive, aerospace, and biomedical areas. AM is based on an additive principle of depositing material layer by layer, offering the beneficial ability to make complexly shaped objects from 3D model data by joining materials. AM technologies open opportunities to make components on-demand for customization, to fabricate structures with complex geometries, to save on material costs, and to compress supply chains by reducing the time to market.

However, AM components are limited to passive structural support material without built-in functionality [1]. Thus, there have been intense efforts to achieve a fully functional 3D structural electronic device by integrating the conductive structures and components (sensors, antennas, or circuits) into AM parts [2]. Among various AM techniques, fused deposition modeling (FDM) shows a high compatibility with electronic components. Espalin et al. reported that a miniature satellite named Cubesat that integrated FDM-produced structures with embedded electronics was successfully launched into space in 2013 [3]. There have been intense studies on integrating electronics into FDM parts [4].

To integrate electronic functions with AM components, the key procedure is to introduce the conductive channel or layer onto the dielectric AM component. Currently, a widely used method is direct writing (DW) by aerosol jet and ink jet printing. In the DW process, the metal lines that serve as electrical interconnections are fabricated by dispensing conductive ink onto the surface through a deposition nozzle. However, the as-deposited pattern usually requires undergoing a 200-°C sintering process to remove the organic solvent and increase the electrical conductivity. This heat treatment may cause thermal damage to the support structures [5].

Moreover, obtaining a good surface finish presents a significant challenge in AM parts. During the ink dispensing process, overspreading usually occurs due to its high flowability [5]. This instability may cause a shortcut or failure of the electric circuit. To achieve better control over the ink flow on the surface, an additional polishing process is always required before the ink dispensing [1,3,6]. 

Recently, laser-induced graphene (LIG) from graphene oxide (GO) films has been reported and applied to produce energy storage devices [7,8]. El-Kady et al. [7] and El-Kady and Kaner [8] applied a two-step method to produce graphene. First, a GO layer was deposited onto the polymer. Then, a laser irradiating process reduced the GO into graphene. Moreover, Lin et al. [9] reported a one-step laser writing method that can directly create graphene on commercial polyimide (PI) tape. It has been shown that laser processing offers a top-to-bottom, noncontact, and highly selective method that produces graphene with high electrical conductivity properties. The conductive lines consisting of LIG are important elements in manufacturing electronic components such as supercapacitors [9], pressure sensors [10], and biosensors [11].

However, the one-step laser writing method for LIG generation in the previous research relies on polyimide as the carbon precursor, which may limit the extensive applications of LIG. Here, we propose a process for direct laser writing onto the additive manufacturing ULTEM 9085. To the best of our knowledge, this is the first study on converting the surface of a 3D printing part into graphene by laser direct writing. The ULTEM 9085 is a new commercial 3D printing material that is certified by the Federal Aviation Administration (FAA) for use in aerospace applications [12]. It is famous for its excellent mechanical properties and chemical resistance. However, it is nonconductive before laser processing. Laser-induced modifications to surface morphology and chemistry were investigated by scanning electron microscopy (SEM), transmission electron microscopy (TEM), and Raman spectroscopy. It was found that porous graphene was produced in areas processed by suitable laser parameters. Conductive LIG lines were produced on the 3D-printed ULTEM sample. This finding creates possibilities for the integration of LIG-made electronics and AM components in a single process.

## 2. Experiments

A high-performance polymer (ULTEM 9085) in filament form was used for the study. The additive manufacturing machine employed for the preparation of the samples was a Fortus 450 mc, supplied by Stratasys. The machine has a large build envelope of 406 × 355 × 406 mm. The process parameters of the AM machine used are shown in Table 1.

**Figure 1:**
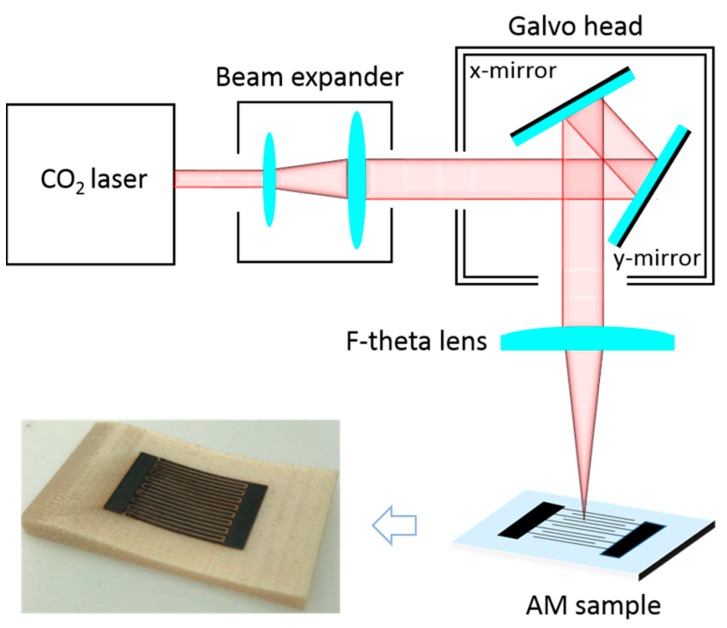
Experimental setup of the laser processing and an image of the laser-induced graphene (LIG) capacitor on the AM part.

The geometry of the specimen was 40 mm × 20 mm × 5 mm, and the building direction was at the edge (X–Z) to achieve a better surface quality. As shown in Figure 1, the AM-printed samples were processed in ambient air using a 10.6-μm CO_2_ laser (SYNRAD FSTi60SFE, Seattle, WA, USA) that emitted linearly polarized continuous wave light with a full power of 60 W. The laser beam was focused and controlled by a pair of Scanlab galvanometer scanners. To maximize the electrical conductivity, the optimized laser scanning speed and hatching density were found to be 20 mm/s and 60 μm, respectively. These two parameters were fixed in the following experiment. The laser power was varied and optimized from 2.4 W to 4.8 W with an interval of 0.3 W to produce the conductive surface. The microstructures of the samples were analyzed using a Jeol JSM 5600-LV scanning electron microscope. The Raman analysis was performed by a Renishaw Invia Raman Microscope with a 514-nm laser source. To investigate electrochemical performance, the cyclic voltammetry, galvanostatic charge-discharge, and cyclability measurements were performed using a VSP-300 workstation. All of the tests were conducted in 1 M of H_2_SO_4_, which served as aqueous electrolytes. 

## 3. Results and Discussion

**Figure 2:**
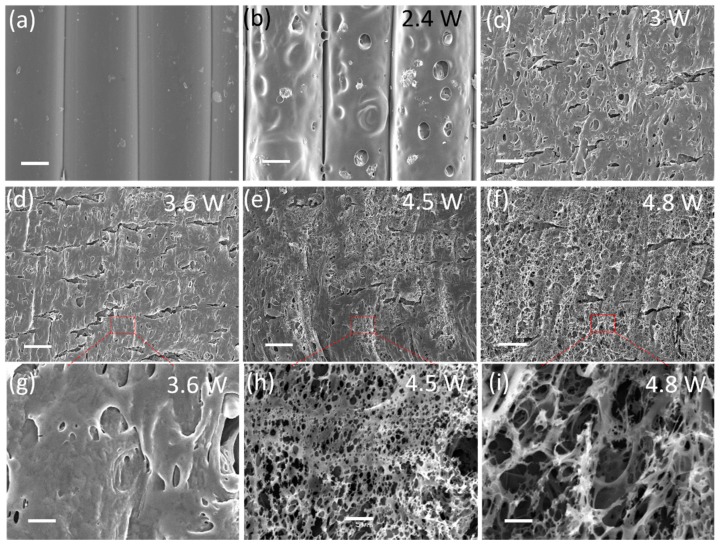
SEM images of the AM parts: Original surface (**a**); surface processed by CO_2_ laser with a power of 2.4 W (**b**), 3 W (**c**), 3.6 W (**d**), 4.5 W (**e**), and 4.8 W (**f**). Scale bar: 100 µm. The SEMs in the third row (**g–i**) are the magnified version of the pictures in the second row, with a scale bar of 5 µm.

Figure 2 shows the microstructure evolution of the ULTEM surface processed by the laser from low power to high power. Figure 2a exhibits an SEM image of the FDM part’s original surface. Due to the nature of the FDM process, microgrooves are observed between the segments of extruded filament. This surface defect is always considered to be a challenge when printing electronics via an aerosol jet process on FDM parts. The grooves may cause uncontrollable spreading of the ink, which results in irregular printed thickness and micropores, which in turn affects the performance of the printed electronics.

After processing by laser with a power of 2.4 W, which was slightly above the damage threshold, as shown in Figure 2b, microdents and bumps were generated by the laser scanning-induced local melting. However, at this low level of laser power, there was no carbon material observed, and the processed area was still nonconductive. 

When the laser power was increased to values above 3 W, as shown in Figure 2c–f, the original surface was completely reformatted and the line grooves diminished, indicating the extruded filament was merged by laser surface processing. At these high laser powers, the ablated areas’ color changed from white to black.

Figure 2h shows that a large area of the LIG film with a porous appearance was obtained at a laser power of 4.5 W. Small holes with diameters of 0.5 to 3 µm could be clearly observed on the thin LIG films. The porous structures may have resulted from the release of gaseous products [9] generated by the massive oxidation. It is worth noting that the porous structures of the LIG exhibiting a large specific area could provide sufficient contact possibilities between electrolytes and the absorption interface, which would strongly increase the efficiency of charge storage for energy storage devices [13]. When the laser power was further increased to 4.8 W, the porous LIG film was replaced by thick foam-like structures, as shown in Figure 2i. This change may have been due to the excessive heat input at the higher laser power, which burned out the extremely thin porous LIG film on the top layer.

**Figure 3:**
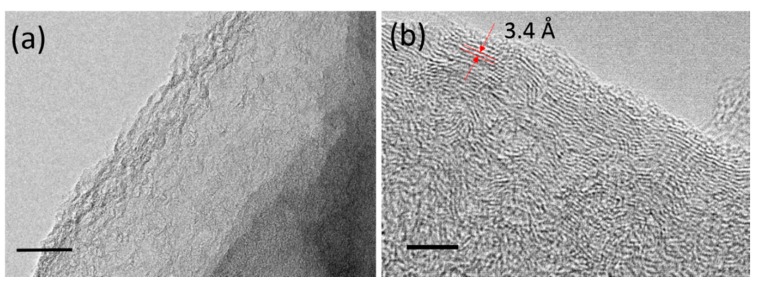
Low-resolution TEM image of the LIG, scale bar: 50 nm (**a**); high-resolution TEM (HRTEM) image taken at the edge of an LIG flake, scale bar: 5 nm (**b**).

The LIG flakes were collected by scratching off the LIG film from the ablated area of the sample surface. These flakes were dispersed in ethanol solution to prepare for TEM analysis. Figure 3 shows TEM images of LIG flakes obtained at a laser power of 4.5 W. Thin graphene layers containing a large amount of wrinkle structures are observed in Figure 3a. The large accessible surface area of nanoscale wrinkles could improve the electrochemical performance of the LIG-made device [9]. Furthermore, Figure 3b exhibits a high-resolution TEM (HRTEM) image taken at the edge of an LIG flake. The observed ripples with a spatial wavelength of 0.34 nm corresponded to the d-spacing of graphene [14].

**Figure 4:**
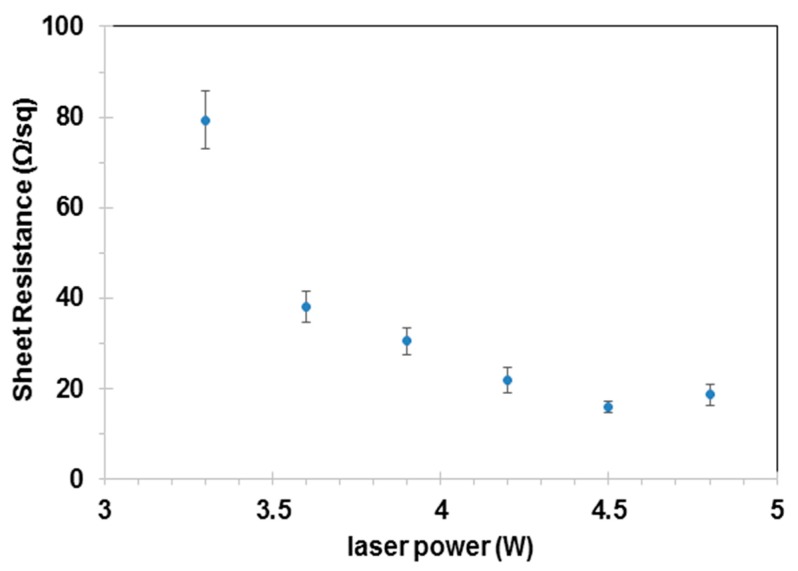
Sheet resistance as a function of laser power.

A 3.5 mm × 6 mm area was processed by varying the laser power to study the sheet resistance (*R_s_*) of the LIG. Each experiment was repeated five times for each combination of laser parameters. In order to maintain good electrical contact, conductive silver epoxy was applied to bond the ablated zone and sheet copper electrode. The ULTEM was originally a nonconductive material. After laser processing with a laser power of 3 W, the ablated area was found to be conductive and had an *R_s_* of 247.1 Ω/sq. Figure 4 plots the variation in sheet resistance of LIG under different CO_2_ laser powers. As the laser power increased from 3.3 W to 4.5 W, the *R_s_* of LIG continuously decreased. At a laser power of 4.5 W, we obtained a highly conductive LIG with an *R_s_* of 15.9 Ω/sq, which was close to the LIG’s *R_s_* value on polyimide [9]. When the laser power was further increased to 4.8 W, the *R_s_*, however, increased. Moreover, it was observed that the LIG started to deform and delaminated from the AM surface at a laser power of 4.8 W or higher. This may have been due to the rapid thermal expansion of LIG, which received excessive heat input at intense laser irradiation.

**Figure 5:**
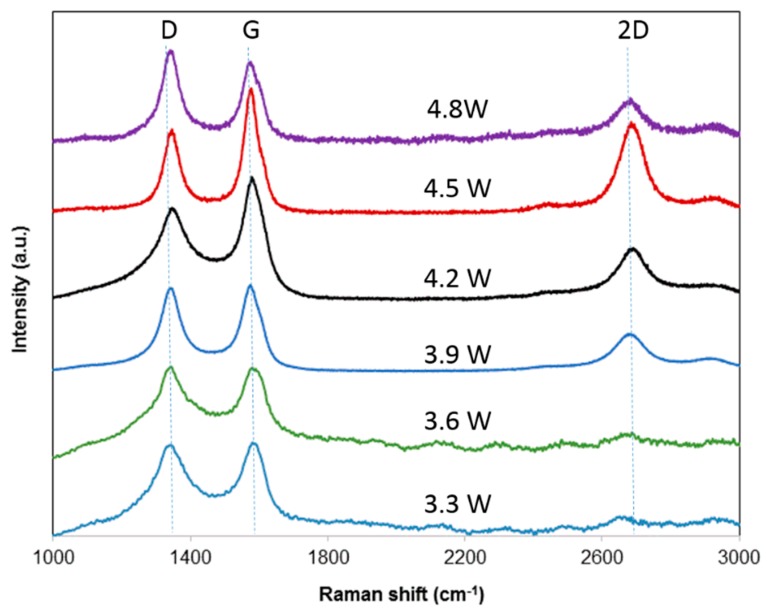
Raman spectrum of LIG obtained at various laser powers.

To further characterize the structural change of the LIG, the ablated areas were investigated by Raman spectroscopy. The Raman spectrum of samples processed by a laser at various powers are shown in Figure 5. The Raman spectrum of the laser-ablated area presented three characteristic peaks of graphenic carbon: a D peak at 1330 cm^−1^ induced by defects or bent sp2 carbon bonds [15], a G peak at 1580 cm^−1^ related to graphite-derived structures [16], and a 2D peak at 2700 cm^−1^, which was the second harmonic of the D band [17].

In particular, it is known that the 2D peak is sensitive, with a number of randomly stacked graphene layers along the *c* axis [18]. The presence of a strong 2D peak can arise from the graphene structures [19] induced by laser processing. This observation is consistent with our finding from the TEM in Figure 3, which showed the generation of graphene at a laser power of 4.5 W. It is reported that the ratio of *I_D_*/*I_G_* is an indicator of crystallization degree for the layered structures [19]. 

With the increasing of laser power from 3.3 W to 4.5 W, the ratio of *I_D_*/*I_G_* decreased to a minimum value of 0.64, suggesting a higher degree of graphene could be obtained at stronger laser powers up to 4.5 W. It is interesting to notice that this laser power (4.5 W) also produced the lowest sheet resistance of 15.9 Ω/sq. Based on this comprehensive study of laser power’s influence on LIG’s electrical resistance and Raman spectrum, it can be concluded that the optimal laser power to produce high-quality graphene is 4.5 W.

In consistent with the trend of electric resistance’s dependence on laser power (in Figure 4), the ratio of *I_D_*/*I_G_* became larger when laser power was further increased to 4.8 W. This change in the ratio of *I_D_*/*I_G_* could be attributed to the oxidation induced by the higher laser power of 4.8 W, which deteriorated the graphene structure.

It is known that the ULTEM 9085 is an amorphous thermoplastic blend consisting of polyetherimide (PEI) with a polycarbonate (PC) copolymer blend incorporated for improving its flowability during the AM process [12]. However, the mixture ratio of PEI to PC is not disclosed, and the chemical composition is kept confidential. It is believed the PEI, which is a typical cross-linked thermosetting plastic, plays a major role in the transformation of the ULTEM into LIG by laser direct writing [20]. The CO_2_ laser, emitting 10.6 µm of light, can directly excite the lattice vibration mode of the PEI, which has a high absorbance (0.5) at a wavelength of 10.6 µm. The rapid thermalization would cause the local temperature to rise above 2500 °C in a time scale of 10^−12^ s [21]. This fast energy input from the laser would break the chemical bond of the polymer chains and result in recombination of the atoms. In other words, the laser processing could create possibilities for a polymer’s structural rearrangement on a molecular scale. Under this circumstance, the sp3 carbon atoms in the surface could be converted to sp2 carbon atoms, indicating the generation of graphene structures.

**Figure 6:**
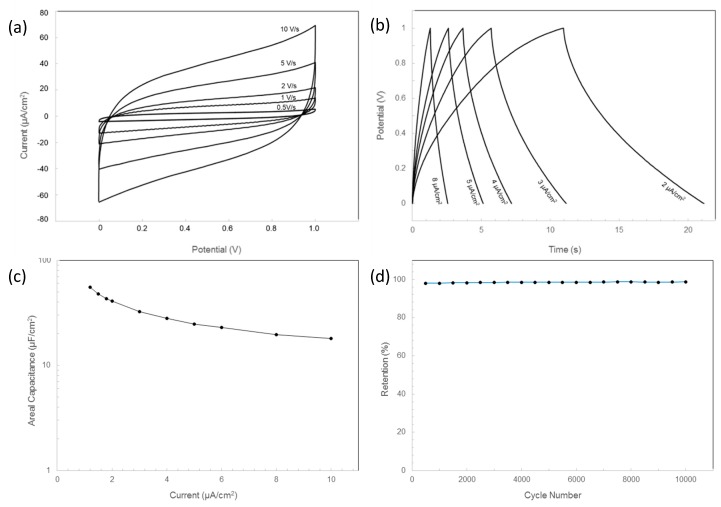
Electrochemical behavior of the LIG capacitor: Cyclic voltammetry (CV) curves of the LIG capacitor at scan rates of 0.5, 1, 2, 5, and 10 V/s (**a**); galvanostatic charge-discharge (GCD) curves at an electric current of 2, 3, 4, 5, and 8 µA/cm^2^ (**b**); *C_A_* calculated from GCD curves as a function of current density (**c**); and cycling performance at a current density of 1.2 µA/cm^2^ (**d**).

It is reported that graphene is a good candidate to produce electronics due to its high electrical conductivity as well as good chemical and mechanical stabilities. To demonstrate LIG’s potential in the application of electronic components, an LIG-made interdigital electrode that serves as a capacitor was fabricated on the AM parts. The image of the capacitor is shown in Figure 1. The laser power applied was 4.5 W, which has been proven to be suitable to produce high-quality graphene. The energy storage ability of the LIG-made capacitor was evaluated by cyclic voltammetry (CV) and galvanostatic charge-discharge (GCD) techniques. Figure 6a exhibits the CV curves of the LIG capacitor at various scan rates. The observed pseudo-rectangular CV curves in Figure 6a suggest the capacitor had good double-layer capacitive behavior [22], which could be confirmed by the triangular-shaped GCD curves at different electrical currents in Figure 6b. Additionally, the GCD results were also used to estimate the capacitance of the sample. The areal capacitance *C_A_* was calculated by Equation (1): [10]

(1) $$C_{A}=\frac{1}{S}\cdot \frac{I\;\cdot t}{V}$$

where *I* is the discharge current, *t* is the discharge time, *S* is the area of the electrode, and *V* is the potential window.

The *C_A_* obtained at different currents is plotted in Figure 6c. With an applied discharge current of 1.2 µA/cm^2^, *C_A_* was found to be 55.2 µF/cm^2^, which was comparable to the previous result (94.3 µF/cm^2^ at 1.25 µA/cm^2^) [18] of porous LIG on a polyimide sample. The relatively smaller *C_A_* in our sample may have been associated with low nitrogen concentrations in the ULTEM [23]. For a better application of the LIG capacitor on the ULTEM, the *C_A_* could be further increased by simply immersing the electrode in HNO_3_ solution to dope the nitrogen into the LIG [23].

The capacitor’s cyclability was evaluated by uninterrupted multiple-cycle charged-discharged testing at a current of 10 µA. Figure 6d shows that the capacitor retained above 98% of initial capacitance after 10,000 cycles, indicating it had excellent cycling stability.

## 4. Conclusions

In this research, a conductive layer was successfully fabricated on ULTEM additive manufacturing parts by CO_2_ laser direct scanning in ambient air. The structural and Raman analyses suggest that the conductive layer consisted of porous graphene, which was converted from the carbon precursor. At a laser power of 4.5 W, the lowest sheet resistance of 15.9 Ω/sq was obtained, indicating the excellent electrical conductivity of LIG. Optimized laser parameters were applied to write an interdigital electrode capacitor on the AM parts. CV, GCD, and cyclability tests confirmed the good electrochemical performance of the LIG capacitor. These results create opportunities to integrate electronic printing with additive manufacturing processes and explore the compatibility of material–deposition–system and manufacturability between two or more printing techniques. 

## Figures and Tables

**Table 1:** Process parameters for the additive manufacturing (AM) machine.

Parameters	Layer Thickness	Contour Width	Air Gap	Raster Angle	Nozzle Sizes
Values	0.254 mm	0.508 mm	0 mm	+45°/−45°	T16
